# Impact of different influenza cultivation conditions on HA *N*-Glycosylation

**DOI:** 10.1186/1753-6561-5-S1-P113

**Published:** 2011-11-22

**Authors:** Jana V Roedig, Erdmann Rapp, Yvonne Genzel, Udo Reichl

**Affiliations:** 1 Max Planck Institute for Dynamics of Complex Technical Systems, Sandtorstraße 1, Magdeburg, Germany; 2 Otto-von-Guericke-University, Chair of Bioprocess Engineering, Magdeburg, Germany

## Background

Influenza virus is a highly contagious human and animal pathogen causing infections of the respiratory track. Prevention such as high standard hygiene and vaccination still represent the best measures for protection. Beside the traditional egg-based influenza vaccine production, numerous cell culture-based processes are currently being established. Due to its ability to induce strong and protective immune responses, the highly abundant glycoprotein hemagglutinin (HA) represents the major component in influenza vaccines. Since variations in *N*-glycosylation of glycoproteins such as HA can alter quality characteristics of antigens, the impact of cell lines and process parameters for vaccine manufacturing needs to be addressed. This study investigates the impact of virus adaptation and different harvest time points on HA *N*-glycosylation. Therefore, the HA of influenza A virus Uruguay/716/2007 (*H3N2*, high growth reassortant), in the following referred to as IVA-Uruguay, was purified and *N*-glycans analyzed by capillary gel electrophoresis with laser-induced fluorescence (CGE-LIF).

## Materials and methods

### Cell culture and virus production

IVA-Uruguay (H3N2, #07/360, NIBSC, South Mimms, UK) was produced in either adherently growing MDCK (No. 84121903) or Vero (No. 88020401) cells purchased from ECACC (Salisbury, UK) . For cell growth GMEM (Invitrogen, #22100-093, Darmstadt, Germany) was supplemented with 5.5 g/L glucose (Roth, #X997.3, Karlsruhe, Germany), 2 g/L peptone (IDG, #MC33, Lancashire, UK), 10 % FCS (Invitrogen, #10270-106) and 4 mg/mL NaHCO_3_ (Roth, #6885.3). Infections were performed in the same medium without addition of FCS but supplemented with trypsin (Invitrogen, #27250-018) at a final concentration of 5 U/mL. Virus was quantified by a hemagglutination assay according to Kalbfuss et al. [1] and is expressed in HAU (log HA/100 µL).

### HA *N*-glycosylation pattern analysis

Virus was harvested and processed for HA *N*-glycosylation pattern analysis according to Schwarzer et al. [2] applying an optimized work-flow [3] and data evaluation [4]. Finally, the samples were separated by CGE-LIF using an ABI PRISM 3100-Avant genetic analyzer (Applied Biosystems, Foster City, California, USA). For data processing and evaluation the x-axis of capillary electropherograms was normalized using an internal standard, resulting in *N*-glycosylation patterns, in which each peak corresponds to at least one distinct *N*-glycan structure. This allowed a direct qualitative comparison regarding *N*-glycan structure presence in different samples. For quantitative comparison, the relative peak height (RPH: the ratio of peak height to the total height of all peaks) was determined for each peak and sample. Low abundant peaks were defined with RPH < 5 %.

## Results

**Figure 1:**
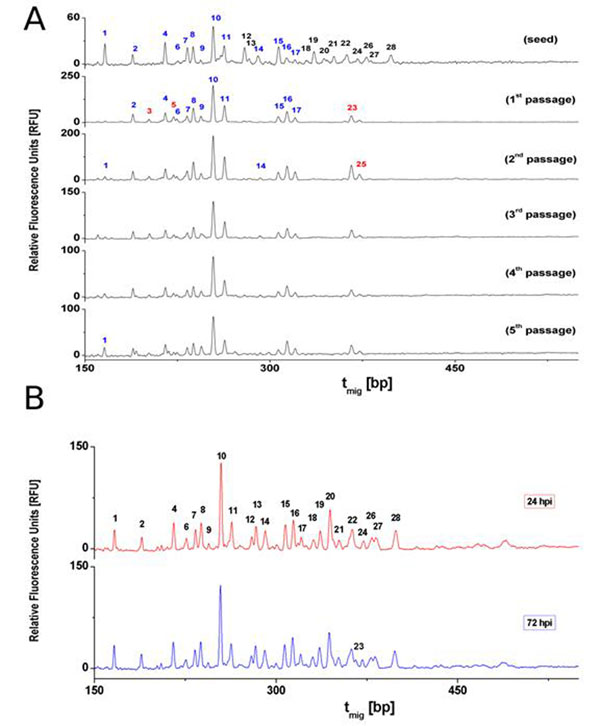
**HA *N*-glycosylation patterns of Influenza A virus Uruguay/716/2007 (*H3N2*) – high growth reassortant.** Relative fluorescence units (RFU) are plotted over the migration time (t_mig_) in base pairs (bp).** (A)** MDCK cell-derived virus (seed) was consecutively passaged in Vero cells (1^st^ passage to 5^th^ passage). Peaks annotated in blue are present in MDCK as well as Vero cell-derived HA; peaks annotated in black are MDCK cell-specific, and red annotation indicates Vero cell-specific peaks. **(B) **MDCK cell-derived virus harvested either 24 or 72 hours post infection (hpi). MDCK cell-specific peaks are annotated (1, 2, 4, 6 – 24, 26-28).

MDCK cell-derived virus seed, exhibiting 2.6 HAU at 24 hours post infection (hpi; data not shown), was used to infect five consecutive passages of Vero cells. Adaptation of the virus to Vero cells resulted in increased virus yields within shorter time frames in the new host system: in the first passage of Vero cells 2.1 HAU were obtained at 96 hpi, whereas in the fifth passage a titer of 2.7 HAU at 72 hpi was reached (data not shown). The HA *N*-glycosylation pattern of the MDCK cell-derived IVA-Uruguay seed exhibited 25 different characteristic peaks in the range of 160 bp to 400 bp. Of these a total number of 11 peaks representing large glycans (275 bp - 400 bp; #12, 13, 18 - 22, 24, 26 - 28) were unique to MDCK cell-derived virus (figure 1A). In contrast, the HA *N*-glycosylation pattern changed significantly with the first passage in Vero cells. Here, 15 different peaks between 150 bp and 380 bp characterize the Vero cell-specific HA *N*-glycosylation pattern. Four peaks (#3, 5, 23 and 25) were unique to Vero cell-derived virus (figure 1A). In comparison to MDCK cell-derived HA, the Vero cell-derived antigen showed a tendency towards smaller glycan structures. The relative abundance of each peak over all Vero passages only varied marginally with standard deviations (SD) ≤ 2.1 % (table 1). The increase in virus titers within shorter time frames suggests increased viral fitness, during adaptation from MDCK to Vero cells. An impact of HA *N*-glycosylation on properties of the virus, i.e. virus replication, has already been descried [8-12]. Interestingly, the glycan pattern stabilized soon after the first passage in Vero cells. This clearly indicates that further increase in HA titer did not depend on changes in the HA *N*-glycosylation pattern.

The impact of harvest time on the HA *N*-glycosylation pattern of MDCK cell-derived IVA-Uruguay is shown in figure 1B. Virus harvested at either 24 hpi or 72 hpi exhibited the 25 different MDCK cell-specific peaks between 160 bp and 400 bp. At 72 hpi one additional, but very low abundant peak was detected (numbered 23). Overall, differences in relative structure abundance were rather small with a maximal difference of 1.7 % RPH (table 1). This indicates that HA of virus particles released in the supernatant is rather stable over the time window relevant for influenza virus production [5,6]].

**Table 1:** **Overview of relative peak heights (RPH) of 28 HA-glycan peaks during virus adaptation from MDCK to Vero cells, and of two harvest time points in a cultivation with MDCK cells.** For virus adaptation, MDCK cell-derived virus seed was consecutively passaged in Vero cells (pass. 1 to pass. 5). Two harvest time points, 24 and 72 hours post infection (hpi), compared by absolute values of RPH and percentage difference |ΔRPH|. Maximal SD and maximal |ΔRPH| are highlighted in bold.

Peak	Virus Adaptation							Time Course		
	seed	pass. 1	pass. 2	pass. 3	pass. 4	pass. 5	pass. 1 to 5	24 hpi	72 hpi	|ΔRPH|
No.	Relative Peak Height (RPH) [%]						SD_RPH_* [%]	RPH [%]		[%]
1	7,8	0,9	1,7	0,9	2,2	4,8	1,6	3,9	4,5	0,6
2	3,9	5,7	4,6	5,1	6,1	4,3	0,7	2,4	2,8	0,4
3	0,0	2,2	1,9	3,4	1,4	1,4	0,8	0	0	0
4	8,1	6,6	5,9	6,1	6,8	6,8	0,4	5,3	5,2	0,1
5	0,0	4,0	2,7	2,5	2,5	1,9	0,8	0	0	0
6	1,4	2,5	1,9	1,5	1,5	1,7	0,4	2,1	1,7	0,4
7	6,2	4,4	4,8	4,1	4,7	4,8	0,3	4,0	3,6	0,4
8	6,5	10,0	9,6	8,1	7,4	7,6	1,2	5,3	5,2	0,1
9	1,7	4,3	3,4	3,4	4,1	5,6	0,9	1,1	1,1	0
10	13,7	25,8	23,5	27,2	28,6	24,3	2,1	17,6	16,3	1,3
11	6,8	11,8	12,3	12,2	11,7	9,5	1,2	5,5	4,9	0,6
12	6,2	0	0	0	0	0	0	2,5	2,5	0
13	2,0	0	0	0	0	0	0	4,6	4,4	0,2
14	3,1	0,6	1,1	1,9	1,3	1,5	0,5	3,7	3,6	0,1
15	6,3	4,1	4,1	4,4	3,8	3,5	0,3	5,0	4,7	0,3
16	2,2	7,7	7,4	7,4	7,8	8,9	0,6	5,9	6,0	0,1
17	1,6	3,3	4,4	3,3	3,1	4,3	0,6	2,3	2,8	0,5
18	1,3	0	0	0	0	0	0	1,9	2,3	0,4
19	4,5	0	0	0	0	0	0	3,6	4,1	0,5
20	2,0	0	0	0	0	0	0	8,0	7,0	1,0
21	2,6	0	0	0	0	0	0	1,7	2,2	0,5
22	3,3	0	0	0	0	0	0	3,9	3,8	0,1
23	0,0	4,7	7,9	6,2	5,0	6,4	1,3	0	1,7	1,7
24	1,9	0	0	0	0	0	0	1,6	1,7	0,1
25	0,0	1,5	3,0	2,3	1,9	2,6	0,6	0	0	0
26	2,4	0	0	0	0	0	0	2,2	2,1	0,1
27	1,1	0	0	0	0	0	0	2,3	2,3	0
28	3,1	0	0	0	0	0	0	3,7	3,4	0,3

* standard deviation (SD_RPH_) of all characteristic peaks in MDCK and Vero cells during virus adaptation

However, there are minor variations in RPH from passage to passage during adaptation and between harvesting time points. Possible explanations are varying ratios either of completely/incompletely processed or of intact/degraded *N*-glycan structures or a combination of both. In 2009, Schwarzer et al. [7] characterized the MDCK cell-derived HA *N*-glycosylation pattern of a *H3N2* influenza virus subtype as a mixture of complex *N*-glycan structures with terminal α- and β-galactose and high mannose type structures. In contrast, the Vero cell-derived HA was characterized by complex *N*-glycans with exclusively terminal β-galactose and structures of the high mannose type. For final evaluation of the results presented here, determination of the *N*-glycan structure of all peaks would be required.

## Conclusion

In this study, the impact of adaptation and harvesting time point on HA *N*-glycosylation of IVA-Uruguay was investigated. So far, it is not clear whether differences in the HA *N*-glycosylation have an impact on immunogenicity or other properties of influenza vaccines. Other factors, e.g. differences in cell culture media, cell density, etc. may also contribute to variations in HA *N*-glycosylation. Nevertheless, monitoring *N*-glycosylation patterns during vaccine production processes allows not only to evaluate antigen quality and the impact of process modifications on lot-to-lot consistency but also to critically assess consequences of unwanted process variations or process failure.

## References

[B1] Kalbfuss B Knochlein A Krober T Reichl U Monitoring influenza virus content in vaccine production: precise assays for the quantitation of hemagglutination and neuraminidase activity Biologicals 2008 36 3 145 161 10.1016/j.biologicals.2007.10.002 18561375

[B2] Schwarzer J Rapp E Reichl U N-glycan analysis by CGE-LIF: profiling influenza A virus hemagglutinin N-glycosylation during vaccine production Electrophoresis 2008 29 20 4203 4214 10.1002/elps.200800042 18925582

[B3] Rödig J Rapp E Hennig R Schwarzer J Reichl U Optimized CGE-LIF-Based Glycan Analysis for High-Throughput Applications Proceedings of the 21st Annual Meeting of the European Society for Animal Cell Technology (ESACT) 2009 Dublin, Ireland: Springer Science+Business Media B.V. in press

[B4] Ruhaak LR Hennig R Huhn C Borowiak M Dolhain RJ Deelder AM Rapp E Wuhrer M Optimized workflow for preparation of APTS-labeled N-glycans allowing high-throughput analysis of human plasma glycomes using 48-channel multiplexed CGE-LIF J Proteome Res 2010 9 12 6655 6664 10.1021/pr100802f 20886907

[B5] Tree JA Richardson C Fooks AR Clegg JC Looby D Comparison of large-scale mammalian cell culture systems with egg culture for the production of influenza virus A vaccine strains Vaccine 2001 19 25-26 3444 3450 10.1016/S0264-410X(01)00053-6 11348709

[B6] Aggarwal K Jing F Maranga L Liu J Bioprocess optimization for cell culture based influenza vaccine production Vaccine 2011 29 17 3320 3328 10.1016/j.vaccine.2011.01.081 21335031

[B7] Schwarzer J Rapp E Hennig R Genzel Y Jordan I Sandig V Reichl U Glycan analysis in cell culture-based influenza vaccine production: influence of host cell line and virus strain on the glycosylation pattern of viral hemagglutinin Vaccine 2009 27 32 4325 4336 10.1016/j.vaccine.2009.04.076 19410619

[B8] Tsuchiya E Sugawara K Hongo S Matsuzaki Y Muraki Y Li ZN Nakamura K Effect of addition of new oligosaccharide chains to the globular head of influenza A/H2N2 virus haemagglutinin on the intracellular transport and biological activities of the molecule J Gen Virol 2002 83 Pt 5 1137 1146 11961269 10.1099/0022-1317-83-5-1137

[B9] Deshpande KL Fried VA Ando M Webster RG Glycosylation affects cleavage of an H5N2 influenza virus hemagglutinin and regulates virulence Proc Natl Acad Sci U S A 1987 84 1 36 40 10.1073/pnas.84.1.36 3467357 PMC304136

[B10] Wang CC Chen JR Tseng YC Hsu CH Hung YF Chen SW Chen CM Khoo KH Cheng TJ Cheng YS Glycans on influenza hemagglutinin affect receptor binding and immune response Proc Natl Acad Sci U S A 2009 106 43 18137 18142 10.1073/pnas.0909696106 19822741 PMC2775302

[B11] Klenk HD Wagner R Heuer D Wolff T Importance of hemagglutinin glycosylation for the biological functions of influenza virus Virus Res 2002 82 1-2 73 75 11885954 10.1016/s0168-1702(01)00389-6

[B12] Wagner R Heuer D Wolff T Herwig A Klenk HD N-Glycans attached to the stem domain of haemagglutinin efficiently regulate influenza A virus replication J Gen Virol 2002 83 Pt 3 601 609 11842255 10.1099/0022-1317-83-3-601

